# Glycoprotein Matrix-Bound Iron Improves Absorption Compared to Ferrous Bisglycinate Chelate and Ferrous Fumarate: A Randomized Crossover Trial

**DOI:** 10.7759/cureus.80224

**Published:** 2025-03-07

**Authors:** Ariane H Secrest, Charlene Norgan Radler, Jaci Kelly, Nikolas Keratsopoulos, Alyssa Faterkowski, Katelyn Kolodziejczyk, Mathis Rollin, Robert Mills, Mandy E Parra, Ralf Jäger, Martin Purpua, Grant M Tinsley, Lem W Taylor

**Affiliations:** 1 Epidemiology and Public Health, University of Mary Hardin-Baylor, Belton, USA; 2 Burnett School of Medicine, Texas Christian University, Fort Worth, USA; 3 Exercise and Sports Science, Human Performance Lab, University of Mary Hardin-Baylor, Belton, USA; 4 Research and Development, Increnovo, LLC, Whitefish Bay, USA; 5 Kinesiology & Sport Management, Texas Tech University, Lubbock, USA; 6 Physiology and Nutrition, University of Mary Hardin-Baylor, Belton, USA

**Keywords:** absorption, iron, postbiotic, supplement, whole-food nutrients

## Abstract

Introduction

The biotransformation of minerals through glycosylation by microorganisms, such as yeast or probiotics, can produce nutrients bound to a food matrix, potentially enhancing their bioavailability. This study aimed to compare the absorption kinetics of iron bound to a glycoprotein matrix (GPM) with those of ferrous bisglycinate chelate (FBC) and ferrous fumarate (FF).

Methods

In a double-blind, crossover design, 17 participants ingested 11 mg of iron in one of three forms: GPM (Pharmachem Innovation, Kearny, NJ, USA), FBC (Ferrochel^®^, Balchem Corp., Montvale, NJ, USA), or FF (FerroPharma Chemicals Ltd, Hungary). Blood samples were collected at baseline and 30-, 60-, 90-, 120-, 180-, 240-, 300-, 360-, 420-, and 480-minutes post-ingestion. Water intake was standardized throughout the protocol, and an iron-free snack was provided at four hours post-ingestion. Pharmacokinetic analysis was performed, with key outcome variables including the incremental area under the concentration vs. time curve (iAUC), maximum concentration (Cmax), and time to maximum concentration (Tmax). The a priori significance level was set at p < 0.05.

Results

Linear mixed-effects models indicated statistically significant effects of the GPM condition for both raw iron concentrations and changes from baseline (p = 0.03). On average, participants had iron concentrations that were 27.1 mcg/dL (95% CI: 2.8 to 51.4) higher after consuming GPM iron compared to the FF reference condition. Changes in iron concentrations from the baseline were 16.6 mcg/dL (95% CI: 1.5 to 31.7) higher after GPM consumption compared to FF. In contrast, iron concentrations and changes in iron levels after FBC consumption did not significantly differ from those observed with FF. Significant effects of time were also observed in both linear mixed-effects models. When expressed as percentage changes from baseline, iron concentrations in the GPM condition were 9.4% to 35.0% higher than FF and 5.9% to 32.6% higher than FBC. Pharmacokinetic analysis revealed a significant effect of condition on the iAUC (p = 0.047), but no significant effects for Cmax (p = 0.15) or Tmax (p = 0.81). Post hoc tests for the iAUC indicated a trend (p = 0.07) for a difference between the GPM and FBC conditions, but no significant differences between GPM and FF (p = 0.17) or FBC and FF (p = 0.75).

Conclusion

These findings suggest that iron bound to a glycoprotein matrix can improve absorption kinetics without any associated side effects. This data could have important implications for addressing iron deficiency or absorption disorders in a variety of populations.

## Introduction

Iron is an essential mineral for human health, supporting critical physiological functions such as oxygen transport, energy metabolism, and cellular division [1,2]. Iron is the most abundant trace mineral in our body [3]. It participates in redox reactions, making it vital for the function of metalloproteins, including hemoglobin and cytochromes [4]. A sufficient daily intake of iron is necessary to maintain iron homeostasis, with maximal bioavailability typically ranging from 14% to 18%, depending on age and sex [5,6].

An estimated two billion people are at risk for iron deficiency, with a global prevalence of 56% among preschool-aged children and 69% among non-pregnant women of reproductive age [7]. Iron deficiency in developing countries is often due to inadequate dietary intake and infections like schistosomiasis, while in developed countries, it is commonly linked to chronic blood loss or conditions that impair intestinal absorption, such as celiac disease. Chronic iron deficiency can lead to symptoms such as fatigue, shortness of breath, brittle nails, delayed wound healing, and dry mouth [8,9]. The World Health Organization recognizes iron deficiency as the leading cause of anemia [10]. Iron deficiency anemia (IDA) can result in impaired cognitive function and adverse pregnancy outcomes [9,11]. Populations at higher risk for IDA include infants, preschool-aged children, menstruating or pregnant women, vegans, frequent blood donors, and the elderly, particularly those with chronic conditions like kidney disease [9]. Treatment typically involves oral iron supplementation, intravenous iron, or red blood cell transfusion in severe cases [9,11].

Iron exists in two forms in the diet: heme and nonheme [1]. Both are absorbed by enterocytes in the duodenum and proximal jejunum. Heme iron is absorbed as an intact molecule, while nonheme iron is absorbed in its reduced (ferrous) form. Transmembrane proteins facilitate iron uptake and efflux into the portal circulation, while ferritin stores iron in enterocytes and hepatocytes. Iron is recycled through the phagocytosis of dying erythrocytes, and circulating iron binds to transferrin for delivery to tissues [12,13]. Iron homeostasis is tightly regulated by the hormone hepcidin, with most iron used by erythrocytes for hemoglobin synthesis [14]. Nonheme iron constitutes about 90% of dietary iron and is found in plant-based foods like nuts, grains, fruits, and vegetables, while heme iron is found in animal products such as red meat, poultry, and fish [1]. Although heme iron represents only about 10% of dietary iron, it is absorbed more efficiently than nonheme iron and may account for up to 40% of total absorbed iron [6]. Nonheme iron absorption is inhibited by polyphenols, phytates, and animal proteins like casein, whey, and egg white. Additionally, calcium reduces the absorption of both heme and nonheme iron. Conversely, nonheme iron absorption is enhanced by co-ingestion of meat, fish, poultry, or dietary vitamin C, which reduces nonheme iron from its ferric (Fe3+) to ferrous (Fe2+) form, which is more readily absorbed [1]. Iron absorption can also be enhanced through fortification with highly bioavailable compounds [6]. Conditions like achlorhydria, atrophic gastritis, Helicobacter pylori infection, celiac disease, small bowel resection, and post-gastrectomy or vagotomy can impair iron absorption due to reduced gastric acidity [12].

Historically, fermentation has been used to preserve foods. The fermentation of carbohydrates produces short-chain fatty acids, which lower the pH and inhibit the growth of pathogenic bacteria. Many different cultures around the world have developed a variety of fermented foods, including sauerkraut (fermented cabbage, Germany), kimchi (fermented vegetables, Korea), miso and natto (fermented soybeans, Japan), tempeh (fermented soy, Indonesia), and fermented dairy products like lassi (India), kefir, and yogurt. Consumption of fermented foods has been linked to promoting a healthy gut microbiome, with increased gut microbiota diversity associated with improved nutrient absorption [15]. A common issue with both organic and inorganic iron supplements is their limited absorption, with about 90% of the iron remaining unabsorbed in the intestines [16]. This excess iron can cause various side effects, including nausea, bloating, vomiting, constipation, diarrhea, abdominal pain, and darkened stools. Incorporating minerals into a glycoprotein matrix (GPM) through double fermentation with yeast and probiotics has been shown to significantly enhance nutrient absorption. For example, a study demonstrated that GPM-bound zinc increased absorption by 40% [17].

This study aimed to compare the bioavailability of GPM-bound iron with two other commonly used supplemental iron forms: ferrous bisglycinate chelate (FBC) and ferrous fumarate (FF). The investigators hypothesized that GPM-bound iron would improve iron absorption and result in higher blood levels after acute ingestion without causing gastrointestinal distress.

This article was previously presented as a poster with published abstract at the International Society of Sports Nutrition National Conference on June 18, 2024.

## Materials and methods

Experimental design

**Figure 1:**
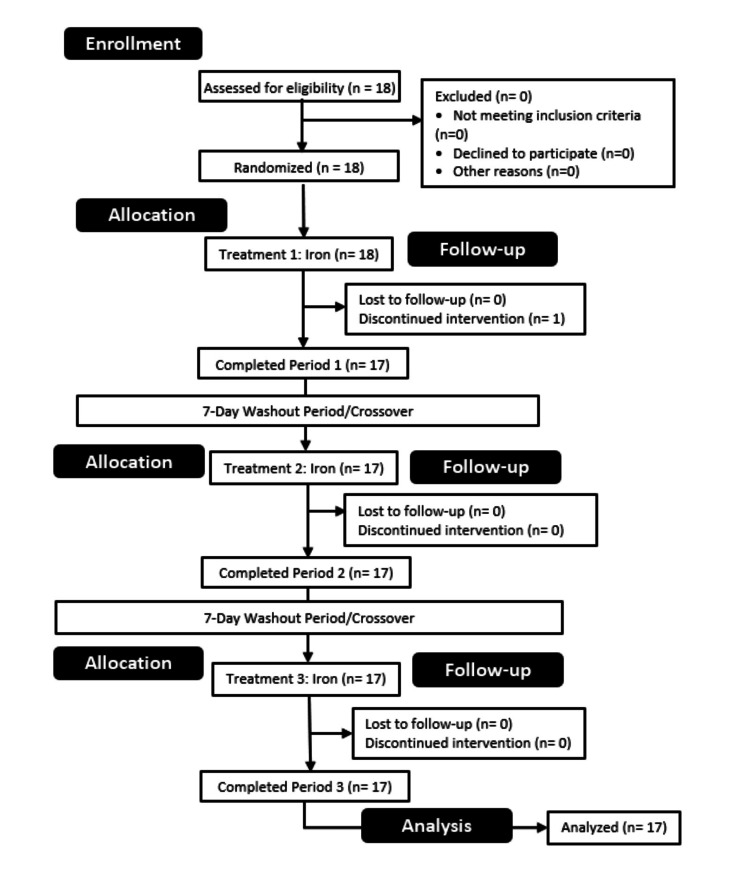
The consortium diagram includes the screening and allocation process for the participants in this trial as well as the numbers regarding those who voluntarily removed themselves from the trial.

A double-blind, randomized crossover study was conducted at the University of Mary Hardin-Baylor (UMHB), Belton, TX, USA, in accordance with the Declaration of Helsinki guidelines. All procedures were approved by the Institutional Review Board of the University of Mary Hardin-Baylor (IRB approval number 259, date of approval: September 27, 2023). Participants visited the Human Performance Lab on three separate occasions after fasting for at least 10 hours and abstaining from caffeine and exercise for 24 hours prior to each session (see Figure 1). Each participant completed an eight-hour absorption trial under three conditions: GPM-bound iron, FBC, and FF. Participants ingested a blinded pill with water, and blood samples were collected prior to ingestion and at 30, 60, 90, 120, 180, 240, 300, 360, 420, and 480 minutes post-ingestion. The order of dietary supplement ingestion was randomized, with a one-week washout period between each testing session.

Participants

The participants in this study were 17 overall healthy men and women, aged 18 to 45 years (22.71 ± 3.7 yrs, 176.2 ± 10.3 cm, 83.0 ± 14.24 kg, 26.6 ± 3.6 kg/m^2^) (see Table 1). To qualify for participation in this study, participants had to have a normal body weight (body mass index (BMI) of 19-24.99 kg/m^2^) and engage in recreational physical activity, as defined by the American College of Sports Medicine guidelines. Participants were not allowed to consume any nutritional supplements known to affect the measures of the current study for at least six weeks prior to participation, including pro-, post- and prebiotics, as well as digestive enzymes. Exclusion criteria included individuals who were currently being treated for or diagnosed with a gastrointestinal, cardiac, respiratory, circulatory, musculoskeletal, metabolic, immune, autoimmune, psychiatric, hematological, neurological, or endocrinological disorder. Also excluded were participants whose body mass had deviated by more than 2% in the previous 30 days, as well as participants who were unwilling to abstain from alcohol, nicotine, and caffeine for 12 hours prior to each visit. Participants were randomized using random.org to first consume one of the three conditions, followed by the other conditions, each after a one-week wash-out period.

**Table 1:** Participant characteristics

	All (n=17)				M (n=9)				F (n=8)			
	Min	Max	Mean	SD	Min	Max	Mean	SD	Min	Max	Mean	SD
Age (y)	19.0	35.0	22.7	3.7	19.0	23.0	21.4	1.1	20.0	35.0	24.1	5.0
Height (cm)	160.8	194.8	176.2	10.3	174.3	194.8	181.4	7.3	160.8	191.5	170.3	10.3
Weight (kg)	56.0	106.0	83.0	14.2	77.4	106.0	91.6	8.9	56.0	94.2	73.3	13.0
Body Mass Index (kg/m^2^)	21.5	33.1	26.6	3.6	23.2	30.4	27.8	2.2	21.5	33.1	25.3	4.4

Experimental protocol

On the day of experimental testing, participants arrived at the laboratory following an overnight fast. All experimental testing sessions occurred between 7 a.m. and 8 a.m. and individual participant start times were repeated on the remaining arms of the crossover design. The occurrence of adverse events was recorded throughout completion of the study visits. Adverse events were collected through spontaneous reporting by the study participants, clinical evaluation or interaction of a research team member with a study participant, and through questionnaires prior to and 480 minutes post-ingestion. The GI Health questionnaires evaluated stomachache, abdominal pain or cramps, bloating, subjective impression of rectal gas excretion and nausea, and ranked side effects on a scale from 0 (no symptoms) to 5 (severe symptoms) [18]. In addition, participants were asked to rank the severity of dizziness, headache, fast or racing heart rate, heart skipping or palpitations, shortness of breath, nervousness, blurred vision, and other unusual or adverse effects on a scale from 0 (none) to 5 (very severe). Participants rested semi-supine for placement of a Teflon catheter into an antecubital vein for multiple blood sampling. The catheter was kept patent by flushing with 2-3 mL of 0.9% sodium chloride. Following baseline sampling, participants ingested their respective supplement with 177 mL of cold water. Thereafter, blood samples were taken at 30, 60, 90, 120, 180, 240, 300, 360, 420, and 480 minutes post-ingestion. Whole blood was collected and transferred into Becton Dickson (BD) 8.5 mL tubes (BD SST Vacutainer, Becton, Dickinson and Company, Franklin Lakes, USA) for obtaining serum and subsequently centrifuged at 1500 g for 15 minutes at room temperature. The resulting refrigerated serum samples were transported to Quest Diagnostic for analysis. Participants ingested the supplement with 177 mL of water immediately after the initial blood collection. The participant then received 59 mL of water every 60 minutes until hour 4, when the participant received an iron-free snack and then again, resumed water consumption at every hour for the remaining four hours. The snack included two servings of Club™ Crackers Snack Stacks (Kellogg Sales Co., USA; 28 g each) and two Member’s Mark™ Light String Cheese Sticks (Sam’s West, Inc., USA; 48 grams). The total caloric intake was 240 calories with 12 g of protein, 20 g of carbohydrates, and 11 g of fat. Subsequently, a one-week wash-out period was implemented before participants were crossed over to the other supplement and repeated the experimental protocol for the two remaining conditions.

Plasma iron analysis

Serum samples were analyzed for plasma iron levels using an FDA-approved method on a Beckman Coulter AU system via spectrometry with Beckman Coulter Iron (total) reagent kit and calibrators (Quest Diagnostics Nichols Institute, Chantilly, VA). Serum samples were analyzed in duplicate and placed on the automated analyzer using manufacturer assay instructions. This Beckman Coulter assay unitizes a variation of the methods described by Schade and colleagues [19] using TPTZ (2,4,6-Tri-(2-pyridyl)-5-triazine) as the chromogen. Transferrin-bound iron dissociates in an acidic medium into free ferric ions and apo-transferrin which are further reduced to the ferrous state using hydrochloric acid and sodium ascorbate. A blue colored complex that can bichromatically (600/800 nm) detect is formed when the ferrous ions react with TPTZ and the absorbance is directly proportional to the serum iron concentration.

Supplementation

All three iron treatments, GPM iron containing 5% Iron (GPM, Pharmachem Innovations, Kearny, NJ, USA), ferrous bisglycinate chelate (FBC, Ferrochel®, Balchem Corp., Montvale, NJ, USA), and ferrous fumarate (FF, FerroPharma Chemicals Ltd, Hungary) contained the equivalent of 18 mg of elemental iron, which represents 100% of the daily value for adults based on FDA guidelines [20]. These treatments were administered in the form of one 0.5 gram uncoated tablet. GPM iron was produced using FF as the iron source, and a carbohydrate and protein substrate. Saccharomyces cerevisiae yeast strains were cultured under heat during the initial phase. After several hours of fermentation, the active nutrient (iron) was added to the cultured broth, initiating the growth phase. The yeast absorbed the iron and incubated it aerobically into its cell structure, forming a matrix around the active nutrient. During the final autolysis phase, enzymes and probiotic bacteria were added at low heat for several hours to complete the digestion process. After this phase, the product was further processed to yield the final powdered glycoprotein-bound active nutrient.

Statistical analysis

Iron concentrations were examined for outliers (i.e., values above Q3 + 1.5 x IQR or below Q1 - 1.5 x IQR). Out of 561 total values (17 participants x 11 time points x 3 conditions), four total outliers (0.7%) were present, with three being baseline values (two in GPM condition and one in FBC condition) and one being a 30-minute post-ingestion value in the FBC condition. These four values were replaced with the mean concentrations within the specified condition and time point. Data were subsequently analyzed in R (version 4.3.1; R statistical software, R Foundation for Statistical Computing, Vienna, Austria) using linear mixed-effects models (nlme package, v. 3.1-162) to determine the relationship between the dependent variable, iron concentrations, and the independent variables condition and time [21]. This approach accommodated the hierarchical structure of the data, where multiple measurements are nested within each subject and are further categorized by experimental conditions and time points. The fixed-effects portion of the model assessed the main effects of condition and time, as well as their interaction. To account for individual variability and potential correlations among repeated measures within the same participant over time, random effects were incorporated into the model. Specifically, a random intercept for each participant was specified, allowing for individual deviations from the overall mean iron level, and a random slope for time within each subject was included to capture individual-specific changes over time. Additionally, correlation between measurements taken at different time points within the same participant and experimental condition were allowed through an autoregressive order 1 (AR(1)) correlation structure. The restricted maximum likelihood (REML) estimation method was utilized to estimate the model parameters, which provides unbiased estimates of fixed effects and more robust estimates of variance components, particularly with smaller sample sizes [22]. Model assumptions were examined through graphical methods (i.e., residuals vs. fitted plots and quantile-quantile plots). Data were visualized using the ggplot2 package (v. 3.5.0) with within-subject error bars for line plots [23,24]. Separate models were fit for raw iron concentrations and, to account for potential differences at baseline, raw changes in iron concentrations from baseline. In both models, the FF group was specified as the reference condition, and time=0 was the reference time point.

For the pharmacokinetic analysis, the incremental area under the concentration vs. time curve (iAUC) was calculated using the method of Brouns et al. [25]. The PKNCA package (v. 0.10.2) was used to establish the maximum observed concentration (Cmax) and time of maximum observed concentration (Tmax) [26]. When statistical assumptions of normality were met, data were analyzed using one-way analysis of variance with repeated measures. Cmax data were analyzed accordingly. However, due to normality violations and outliers, iAUC values were analyzed by the non-parametric Friedman test, with Wilcoxon signed-rank post hoc tests. Due to the nature of the data, Tmax values were also analyzed using the non-parametric Friedman test. These analyses were performed using the rstatix package (v. 0.7.2) [27]. For outcomes analyzed using one-way analysis of variance, Cohen’s d effect sizes were calculated. For outcomes analyzed using the Friedman test, Kendall’s W effect sizes were calculated. For all tests, statistical significance was accepted at p < 0.05, and p-values between 0.05 and 0.1 were considered trends.

## Results

Serum iron concentrations

**Figure 2:**
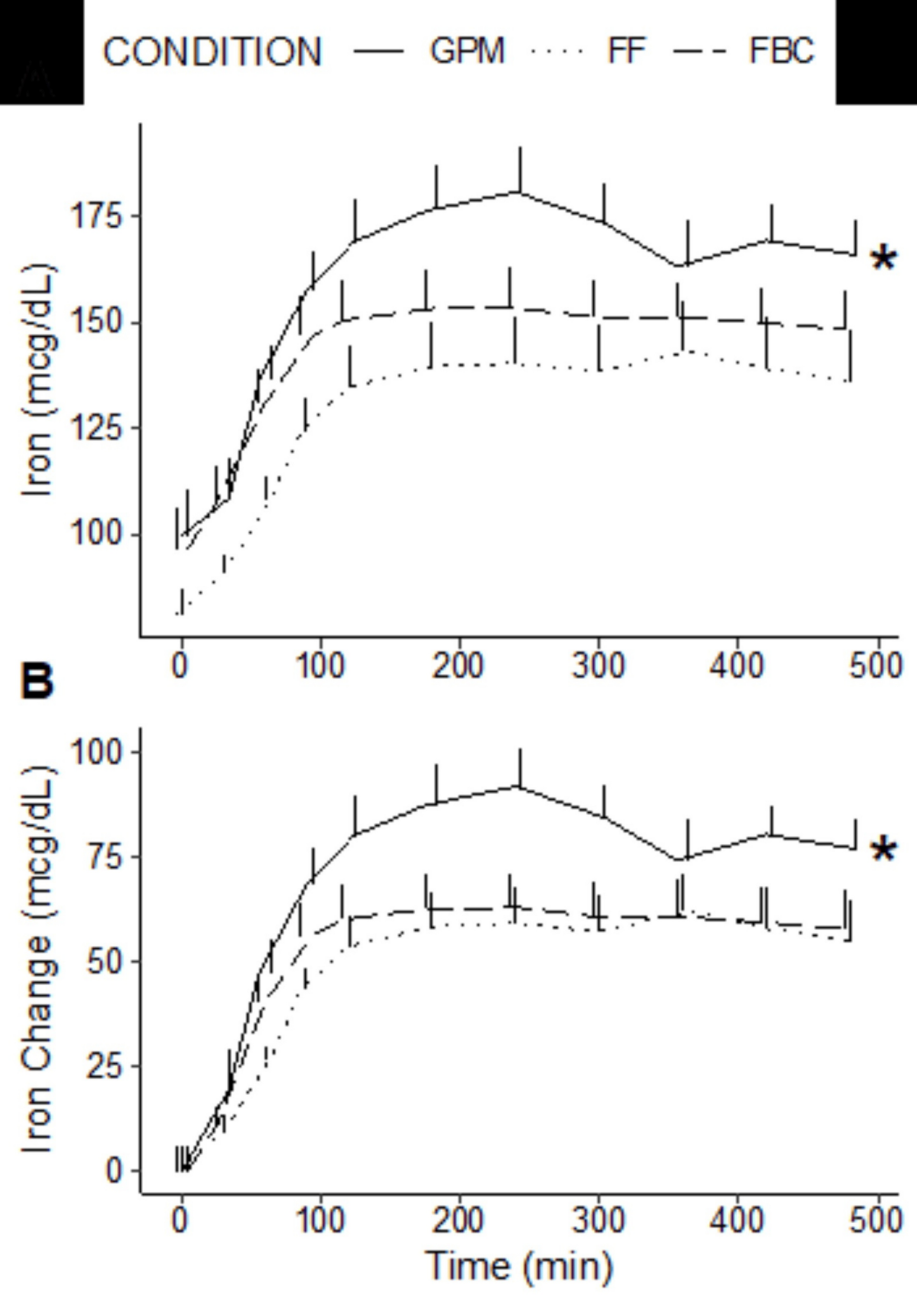
Iron concentrations. Raw iron concentrations (A) and raw changes in iron concentrations (B) are displayed. In linear mixed-effects models for each outcome, there was a statistically significant effect of the GPM condition (*), indicating higher iron concentrations in GPM as compared to the reference model. GPM: glycoprotein matrix-bound iron; FBC: ferrous bisglycinate chelate; FF: ferrous fumarate

Linear mixed effects model terms indicated that there were statistically significant effects of the GPM condition, both for raw iron concentrations and changes from the baseline (p=0.03 for each; Figure 2, Table 2). On average, participants had iron concentrations that were 27.1 (95% CI: 2.8 to 51.4) mcg/dL higher after GPM consumption as compared to the reference condition (i.e., FF consumption). Additionally, changes in iron concentrations from the baseline were 16.6 (95% CI: 1.5 to 31.7) mcg/dL higher after GPM consumption as compared to the reference condition. In contrast, iron concentrations and iron changes after FBC consumption did not significantly differ from the reference condition. Significant effects of time were also observed in both linear mixed effects models. The magnitude indicated that, on average, each one-minute interval corresponded to an increase in 0.1 (95% CI: 0.05 to 0.15) mcg/dL in iron concentrations, holding other terms constant. When expressed as percent changes in iron concentrations from the baseline, values in the GPM condition were 9.4 to 35.0% higher than FF and 5.9 to 32.6% higher than FBC (see Table 2).

**Table 2:** Differences in percent changes in iron concentrations from the baseline. For each comparison of conditions, the difference in the percent change in iron concentrations from the baseline is displayed. GPM: glycoprotein matrix-bound iron; FBC: ferrous bisglycinate chelate; FF: ferrous fumarate

Time (min)	GPM vs. FF	GPM vs. FBC	FF vs. FBC
0	0	0	0
30	9.4	6.3	-3.1
60	22.3	5.9	-16.4
90	28.7	11.2	-17.5
120	30.2	21.4	-8.8
180	32.7	28.9	-3.8
240	35.0	32.6	-2.4
300	29.8	26.2	-3.6
360	14.5	14.9	0.4
420	25.1	21.3	-3.8
480	24.6	17.5	-7.2

Pharmacokinetics

**Figure 3:**
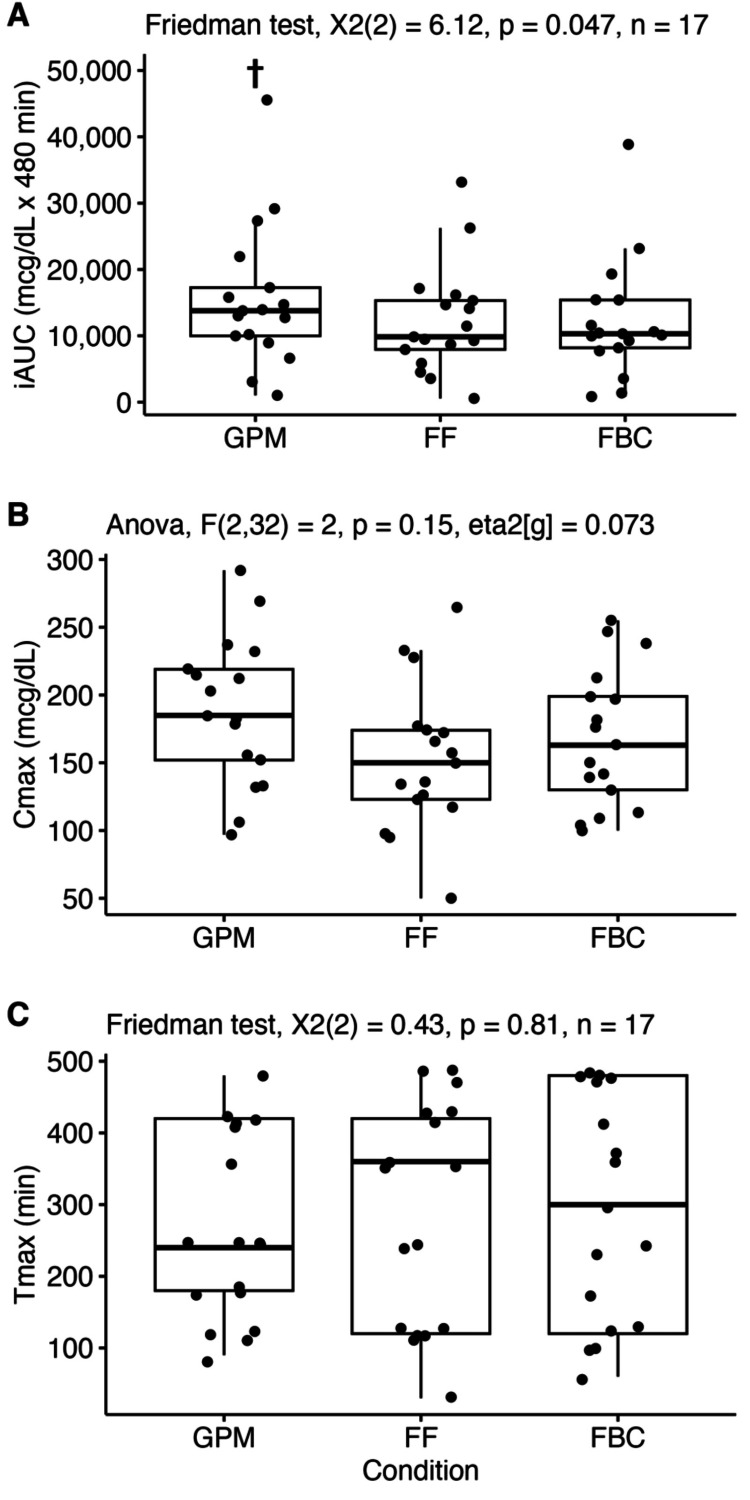
Pharmacokinetic analysis. Analysis of the incremental area under the curve (iAUC), maximal observed concentration (Cmax), and time of maximal observed concentration (Tmax) are displayed. A statistically significant effect of condition was observed for iAUC, with post hoc tests indicating a trend (p=0.07, indicated by †) for a difference between iAUC in the GPM and FBC conditions, without differences between GPM and FF (p=0.17) or FBC and FF (p=0.75). GPM: glycoprotein matrix-bound iron; FBC: ferrous bisglycinate chelate; FF: ferrous fumarate

In the pharmacokinetic analysis, a significant effect of condition on the iAUC was observed (p=0.047; see Figure 3 and Table 3), without statistically significant effects of condition for Cmax (p=0.15) or Tmax (p=0.81). Post hoc tests indicated a trend (p=0.07) for a difference between the iAUC in the GPM and FBC conditions, without differences between GPM and FF (p=0.17) or FBC and FF (p=0.75). For the iAUC, the magnitude of the effect size for the GPM condition was “moderate” as compared to both FBC and FF (see Table 4).

**Table 3:** Pharmacokinetic results iAUC and Tmax were analyzed using the Friedman test due to statistical assumption violations for one-way repeated measures ANOVA or due to the nature of the data. Cmax was analyzed using one-way repeated measures ANOVA. iAUC: incremental area under the curve; Cmax: maximal observed concentration; Tmax: time of maximal observed concentration; GPM: glycoprotein matrix-bound iron; FBC: ferrous bisglycinate chelate; FF: ferrous fumarate; IQR: interquartile range

Outcome	Condition	Mean	SD	Median	IQR	p
iAUC (mcg/dL x 480 min)	GPM	15,594	10,720	13,778	7,275	0.047
	FF	12,237	8,133	9,844	7,373	
	FBC	12,127	8,967	10,313	7,200	
Cmax (mcg/dL)	GPM	188	55	185	67	0.152
	FF	153	54	150	51	
	FBC	168	51	163	69	
Tmax (min)	GPM	263	130	240	240	0.807
	FF	288	156	360	300	
	FBC	293	161	300	360	

**Table 4:** Effect sizes ^1^For outcomes analyzed using one-way analysis of variance (Cmax), Cohen’s d effect sizes are presented. For outcomes analyzed using the Friedman test (iAUC and Tmax), Kendall’s W effect sizes are presented. ^2^Magnitudes are generated by the rstatix R package (v. 0.7.2) [26]. iAUC: incremental area under the curve; Cmax: maximal observed concentration; Tmax: time of maximal observed concentration; GPM: glycoprotein matrix-bound iron; FBC: ferrous bisglycinate chelate; FF: ferrous fumarate

Outcome	Comparison	Effect Size^1^	Magnitude^2^
iAUC	GPM vs. FF	0.34	Moderate
	GPM vs. FBC	0.44	Moderate
	FF vs. FBC	0.09	Small
Cmax	GPM vs. FF	0.45	Small
	GPM vs. FBC	-0.19	Negligible
	FF vs. FBC	0.33	Small
Tmax	GPM vs. FF	0.11	Small
	GPM vs. FBC	0.19	Small
	FF vs. FBC	0.11	Small

Side effect monitoring

No side effects were reported. Specifically, for all side effects (nausea, abdominal pain, bloating, gas, diarrhea, dizziness, headache, racing heart, palpitations, shortness of breath, nervousness, and blurred vision) at all time points in each condition, all participants provided ratings of zero.

## Discussion

In the current study, GPM-bound iron demonstrated increased absorption, leading to higher blood levels after ingestion, with no reports of gastrointestinal distress. Given the essential role of iron in a variety of vital bodily functions, including the transportation of oxygen, metabolic energy, immunity, and DNA replication and repair [1], the number of individuals with deficient iron levels is alarming. Approximately two billion people around the world are affected by iron deficiency and IDA, with iron deficiency reported as the underlying cause of 30 million disability-adjusted life-years (DALYs) [28,29]. Iron deficiency can impair cognitive and physical development in infants and adversely affect fetal growth and gestational length in pregnant women [30]. Given iron’s crucial role in supporting various vital functions across the lifespan, it is critical to find strategies to reduce the number of individuals worldwide who are impacted by iron deficiency. One promising approach, as highlighted in the current research, is to develop mechanisms that maximize iron bioavailability and absorption.

Reducing the risk of IDA largely depends on improving iron bioavailability [1]. Several dietary factors are known to influence iron bioavailability with the most notable enhancers being the co-ingestion of ascorbic acid, targeting animal-based foods as the source for iron, and iron fortification [1,6]. Over the past few decades, methods to improve iron bioavailability have been subject to increasing research [31-33]. Recent studies have explored additional potential enhancers, such as the administration of an extract of black pepper, which has been shown to enhance iron bioavailability [34,35]. The impact of fermentation to enhance the iron bioavailability has also been demonstrated as effective in multiple studies [36-38]. 

Our study showed that GPM-bound iron significantly increased iron concentrations compared to FF, by up to 35%, and FBC, by up to 33%, confirming the previously observed improvement in mineral absorption using GPM technology [15]. The results are particularly meaningful, as FBC has previously been reported to have at least twice the bioavailability of conventional iron salts and has been associated with fever-related adverse events [39-42]. This data has potential implications as a means of increasing hemoglobin and ferritin concentrations, especially in groups at risk of iron deficiency such as children, pregnant women, and athletes.

GPM nutrients are derived from a nutrient-rich broth that is cultured and bio-transformed through glycosylation by microorganisms, including probiotics and yeast. The incorporation of inorganic minerals into the GPM food matrix results in a slower, sustained release of zinc compared to inorganic mineral salts [15]. In our study, we compared GPM iron with organic salts (such as fumarate) and amino acid chelates (like bisglycinate) and found that GPM iron did not exhibit a slower absorption profile. Given that gastrointestinal symptoms, including constipation, nausea, and diarrhea, are commonly associated with iron intake [43], it is important to note that all three forms were well tolerated with no gastrointestinal discomfort reported. Additionally, when participants were asked about other side effects, such as headaches, no adverse events were reported in any treatment group.

GPM contains postbiotics, and repeated ingestion of GPM iron may further enhance absorption by improving gut microbiota composition, which in turn can boost nutrient absorption. The International Scientific Association of Probiotics and Prebiotics (ISAPP) defines a postbiotic as "a preparation of inanimate microorganisms and/or their components that confers a health benefit" [44]. Postbiotics must include intact cells or cell fragments and be produced from bacteria through a deliberate killing process (e.g., heat, radiation, or lysis). GPMs are manufactured through a double-fermentation process involving yeast and probiotics, with the probiotics undergoing heat treatment to ensure their inactivation. In addition to enhancing nutrient absorption, postbiotics like heat-killed Lacticaseibacillus rhamnosus CRL1505 also offer immune benefits [45]. Furthermore, postbiotics have been shown to support mood and reduce fatigue [46].

The strengths of this study include the crossover design, which allows for the removal of the inter-subject variability across groups and reduces the impact of covariates [47]. As the study was double-blinded, participants were unaware of which supplement they were given, as were the study investigators, which minimizes the risk of introducing potential bias. As FBC has previously been shown to demonstrate improved bioavailability over FF [48], this strengthens the results of the current study as the GPM condition was found to have greater iron serum levels when compared to the FBC and FF conditions.

This trial does present some possible limitations to the experimental design and findings. This is a single-dose study, and it is not known how long-term consumption could influence the absorption kinetics in a single dose as a result of chronic exposure or cumulative ingestion effects. Initially, no food was consumed in the first half of this trial; thus, possible interactions with concurrent food intake could be present, altering absorption rates. The duration of measurement also presents a possible limitation as absorption likely continues past the eight-hour window studied here. Data presented here show absorption differences using tablets; thus, future studies should investigate how different delivery formats (e.g. capsule, suspension, etc.) impact absorption rates. This study used healthy individuals and investigated subpopulations that are anemic or women during their menstrual cycle to check if initially lower iron concentration impact iron absorption would be warranted. Another limitation of this study is the small/moderate sample size; thus, more research is needed on this topic. Additionally, future research should evaluate if bioavailability is influenced by the dose of iron.

## Conclusions

Iron bound to a GPM significantly increases absorption compared to two commonly consumed and readily available forms of iron supplementation. This effect is observed in the peak concentration of serum iron following acute ingestion, where it outperforms both FBC and FF. GPM iron was well tolerated without any gastrointestinal tract distress.

## Appendices

Gastrointestinal symptoms questionnaire

https://docs.google.com/document/d/12BZRsjLQGBH1ksgXj6iy-6UA_u5ghb45/edit?usp=sharing&ouid=116138793772992713231&rtpof=true&sd=true
